# Research Trends in Advanced Glycation End Products and Obesity: Bibliometric Analysis

**DOI:** 10.3390/nu14245255

**Published:** 2022-12-09

**Authors:** Patricia Budihartanti Liman, Karina Shasri Anastasya, Nabila Maudy Salma, Yenny Yenny, Meutia Atika Faradilla

**Affiliations:** 1 Department of Nutrition, Faculty of Medicine, Universitas Trisakti, Jakarta 11440, Indonesia; 2 Nutrition Study Center, Faculty of Medicine, Universitas Trisakti, Jakarta 11440, Indonesia; 3 Ciputra Hospital Tangerang, Tangerang 15710, Indonesia; 4 Department of Anatomy, Faculty of Medicine, Universitas Trisakti, Jakarta 11440, Indonesia; 5 Department of Pharmacology and Medical Pharmacy, Faculty of Medicine, Universitas Trisakti, Jakarta 11440, Indonesia; 6 Department of Biochemistry, Faculty of Medicine, Universitas Trisakti, Jakarta 11440, Indonesia

**Keywords:** advanced glycation end products, bibliometric analysis, obesity, VOSviewer

## Abstract

The aim of this study was to conduct a bibliometric analysis of the scientific articles on advanced glycation end products (AGEs) and obesity. English-language journal articles about AGEs and obesity were retrieved from the Scopus database. The OpenRefine application was used for data cleaning, the VOSviewer software program for analysis of the trends of year of publication, country, institution, journal, authors, references, and keywords. Microsoft Excel and Tableau Public were applied for the visualizing of the publication trends. Data collection was performed on 3 February 2022, from a total of 1170 documents. The Mann–Whitney test and Spearman test with software SPSS ver.28.0.1.1. were used to assess the relation between open access journal statuses, years of publications, and CiteScore. The results of the study showed that there was an increase in studies on processed foods, including AGEs and obesity. The United States was the country with the largest contribution in this field, with the highest number of citations. The *Nutrients* journal published the largest number of articles on this topic, particularly in the last two years. The present focus of the studies is on ultra-processed foods. The open access journals have younger medians of the year of publication and higher medians for number of citations than do closed access journals (*p* < 0.001 and *p* < 0.05, respectively). A strong negative association was seen between CiteScore and the year of publication (r = −0.64 [95% CI: −0.67, −0.60]), *p* < 0.001. We present this bibliometric analysis to furnish the most recent data on the description, visualization, and analysis of AGEs and obesity.

## 1. Introduction

Obesity is a global problem that is still unsolved. This may also be seen in Indonesia from the yearly increases in the prevalence of obesity [1]. The shift in lifestyle as a result of globalization and modernization leads to an increase in sedentary activities and the consumption of unhealthy foods such as processed foods, and in overnutrition [2,3]. This lifestyle, in conjunction with smoking [4] and hyperglycaemia [5], is known to increase the level of advanced glycation end products (AGEs) in the body, which subsequently cause disease complications [6].

Publications about AGEs started to develop with the discovery of AGEs in the body in connection with diabetes [7]. The increase in publications became more noticeable after the breakthrough discovery of AGEs in foods [8] that are consumed daily [9,10,11,12,13]. There were also other discoveries, such as the effect of heating [14], pH [15], and the duration of food storage [16], all of which may be changed to reduce the level of AGEs in foods. In spite of this, in general the studies demonstrated that processed foods and high fat foods have higher AGE levels than unprocessed food. These studies, initially concerned only with the content of salt, sugar, fats, and fiber in foods [17], added to the knowledge of processed foods to include other active substances, namely AGEs [18]. The available studies show that foods that are high in AGEs have the greatest effect on the level of AGEs in the body, apart from cigarettes and endogenously formed AGEs [19,20]. The high AGE content in processed foods also brought about changes in the studies in this field. This is apparent from the fact that the keyword “food industry” occupies the fourth highest rank in publications on foods [21].

It is well known that study results, ideas, and discussions are shared by being published as scientific articles [22]. For the objective and subjective retrospective mapping of the trends in an abundance of publications possibly numbering in the thousands, bibliometric analysis is required. Bibliometric studies may become the foundations for overviewing the available study topics, searching for knowledge gaps, finding ideas for studies that have never been conducted previously or are still in small numbers, and for determining the position of the research contribution [23], such as performance analysis and subjective analysis. Scopus is one of the international commercial scientific publishers of global reputation, whose database is regarded as being reliable and comprehensive for use as a bibliographic database and even superior to other databases [22].

The publication of journal articles on AGEs in obesity was first recorded in the Scopus database at the end of the twentieth century [7], after which studies on AGEs and their complications increased annually with the various developments [24]. One study showed that AGEs have become the fifth highest-ranking topic in studies on diabetic kidney disease [25]. However, there are no systematic analytical studies on the publication trends of AGEs with obesity.

This study presents the publication trends on AGEs in obesity using bibliometric analysis by means of VOSviewer. The aim of this study was to obtain visual and quantitative information from the available scientific publications of AGE studies, namely for determining the publication trends of these studies in the Scopus database, for the mapping of publications on AGEs by researcher, affiliated institution, and country of publication, and for determining the degree of collaboration between researchers.

## 2. Materials and Methods

**Figure 1:**
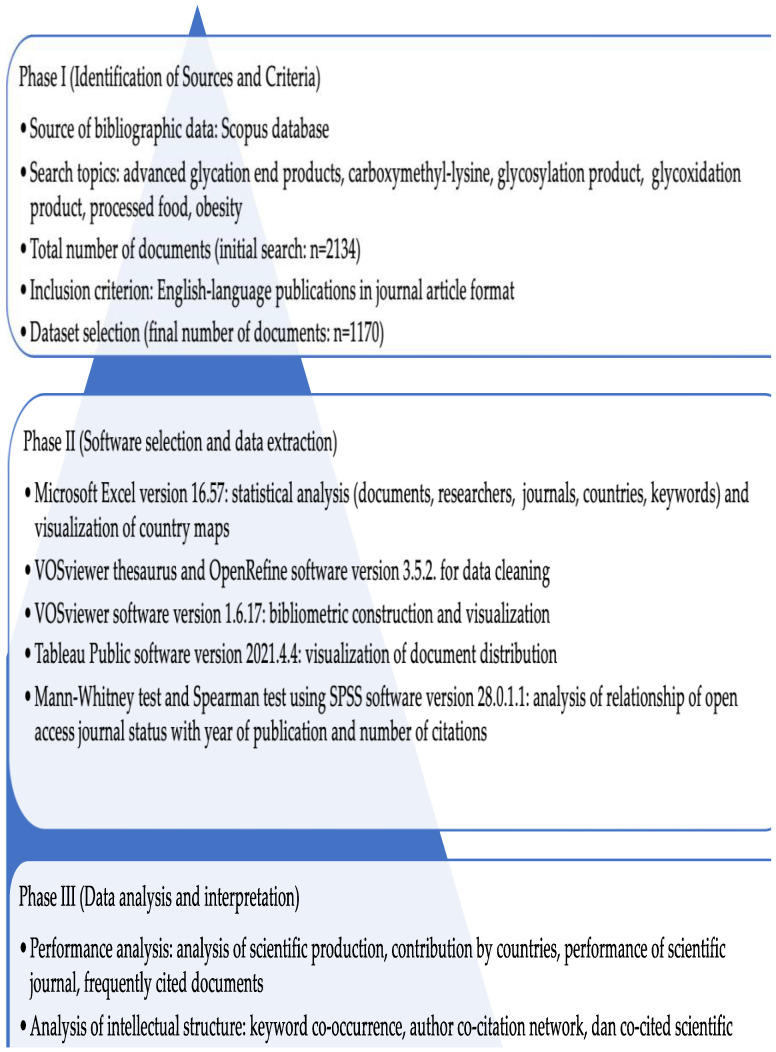
Phases of methodology and analysis used in this study.

The methodology of this study may be divided into three phases, i.e., the identification of the sources and criteria for the data search, software selection and data extraction, and finally data analysis and interpretation (Figure 1).

### 2.1. Phase I—Identification of Data Sources and Search Criteria

The investigators tried to identify publications on AGEs in an objective manner: to analyze the distribution of scientific productions with regard to researchers, institution, or country, and to evaluate the structure and evolution of the field of AGE research. Our study used documents obtained from the Scopus database, which is a database of peer-reviewed journals that allows bibliometric studies in analysis and visualization, supporting the scope of journals on AGEs.

Data collection was conducted on 3 February 2022, using search terms associated with AGEs and obesity in the titles and keywords, in the following format: (TITLE-ABS-KEY (“advanced glycation end product*” OR “carboxymethyl-lysine” OR “glycosylation product” OR “*glycoxidation product” OR “processed food”) AND TITLE-ABS-KEY (“obesity”)) AND (LIMIT-TO (PUBSTAGE, “final”)) AND (LIMIT-TO (DOCTYPE, “ar”)) AND (LIMIT-TO (LANGUAGE, “English”)) AND (LIMIT-TO (SRCTYPE, “j”)). On the initial search, the researchers found 2134 documents.

The inclusion criteria were then confined to the final publications of articles in English-language journals and excluding review articles, book chapters, conference articles, books, notes, letters, errata, short surveys, and others. However, our study did not place limits on the year of publication. The final number of documents in this phase was 1170 documents.

### 2.2. Phase 2—Software Selection and Data Extraction

The mined data from Scopus were exported as comma-separated values in Microsoft Excel Home and Student 2019 edition version 16.57. The information that was downloaded for each publication was data on authors, abstract, keywords, document titles, year, number of citations, bibliographic information, funding details, open access journal label, and references. The values of Source Normalized Impact per Paper (SNIP) were taken from the article of Bajjou M.S. [26]. The statistics of Q-ranking, SCImago Journal & Country Rank (SJR), and h-index were taken from the website https://www.scimagojr.com/journalrank.php (accessed on 29 March 2022). The VOSviewer software version 1.6.17, from Leiden University, Leiden, Netherlands, was used for bibliometric construction and graphics. In data cleaning, the spelling of words with the same meaning was uniformly formatted using the OpenRefine software version 3.5.2 and thesaurus text (thesaurus table). Non-relevant terms were manually removed. Visualization of figures, graphics, and tables was performed by means of Tableau Public version 2021.4.4. and Microsoft Excel. The Mann–Whitney test in SPSS software version 28.0.1.1 was used to analyze the relationship of the status of open access journals with year of publication and number of citations.

### 2.3. Phase 3—Data Analysis and Interpretation

Data were analyzed and interpreted objectively and subjectively, and subsequently underwent structured and documented evaluation. Performance analysis was performed objectively, i.e., by evaluating the trends for year of publication, country, journal, and greatest number of documents and citations. Scientific mapping was performed subjectively, namely by evaluating keywords, author networks, and scientific journal co-citation networks. We also analyzed the relation of open access journal status, including years of publications and CiteScore.

## 3. Results

### 3.1. Performance Analysis

#### 3.1.1. Distribution of Publication by Year

**Figure 2:**
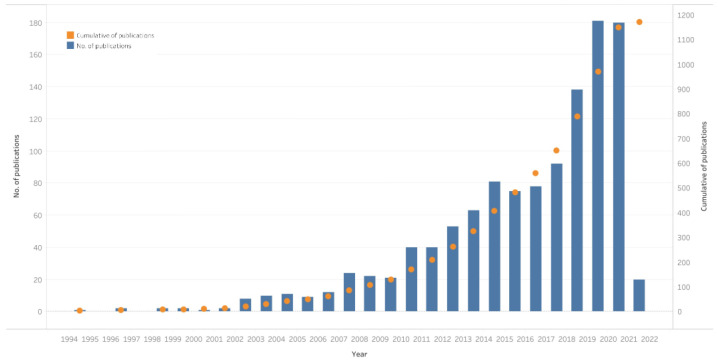
Distribution of publications on AGEs in obesity for 1995–2022.

There were 1170 publications of studies on AGEs in processed foods and obesity, in the form of articles in English-language journals from 1988 to 2022 in the Scopus database (Table S1). Figure 2 shows that there were yearly increases in the number of research publications. There was a considerably sharp increase in 2003 that triggered a surge in the number of publications in the following years.

The publication data in the graph for 2022 seem lower than 2021. This is because data are taken until 3 January 2022, so they do not reflect the publication data in one whole year. However, if it is simulated with the same publication rate per month, the estimated publication figure reaches 200 articles in 2022, or exceeds the number of publications in 2021.

#### 3.1.2. Contributions by Country and Region

**Figure 3:**
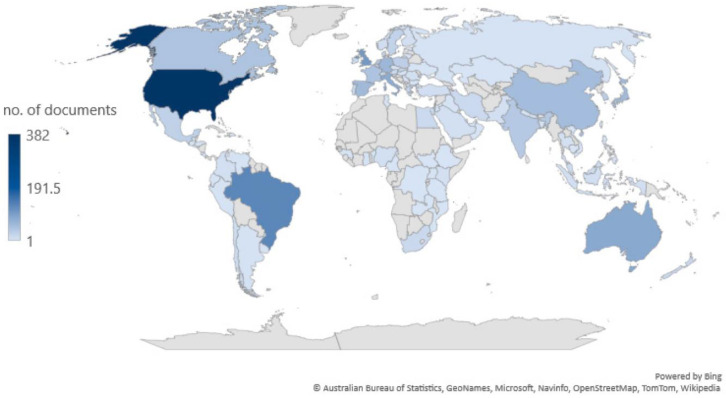
Distribution of publications by country.

Bibliometric coupling analysis was used to evaluate the number of documents from the researchers’ country of origin. There were 112 countries that met the minimum criterion of one document. The distribution of the countries involved in the publications may be seen in Figure 3. The United States predominates in the number of publications on AGEs and obesity with 382 documents.

Table 1 shows the top 15 countries based on the number of documents in the field of AGEs and obesity using VOSviewer. Brazil, the UK, and Australia are the countries contributing the largest number of publications after the United States. Regarding the number of cited publications, the US also occupies the highest rank with 19,362 citations, while the UK and Italy occupy the second and third ranks with 4203 and 3988 citations, respectively. The developed countries that are equated with a high consumption of fast foods and processed foods dominate the number of documents in this field.

Table 2 shows the three topmost ranking countries per cluster based on the number of documents. In Cluster 2 (coded green), the three highest ranking countries from Asia among the fifteen countries with the highest number of documents are Japan, China, and India with 57, 53, and 37 documents, respectively. European countries are dominated by the UK (102) in Cluster 10. There are two other countries in Cluster 10, namely Oman and South Sudan. The UK has strong links with the US (4926), Brazil (2989), and Australia (1665). Oman and South Sudan collaborate with the UK and the US, having links of 80 and 78, respectively, which are the lowest collaboration levels if compared with the other countries in this field. Lontchi-Yimagou, E., from Cameroon in Cluster 5, published an article on AGE receptors as inflammatory biomarkers in the body that may be associated with insulin resistance and the complications of obesity. This publication contributes much to the knowledge of the probable inflammatory pathway, which may have increased the number of citations for this document. The collaboration of the authors from Cameroon with other authors from the United States, the Netherlands, Australia, and South Africa increase the number of links to 26, if compared with Oman (4) and South Sudan (4). This is also seen in Cluster 7, where Cyprus contributes one document on dietary quality, particularly in connection with ultra-processed foods, in subjects participating in the European I.Family Study. In the latter study, there are 17 authors involved in writing documents with authors from Cyprus with 12 affiliations and 9 countries with links to Cyprus (47 links), which is higher than for authors from Cameroon, with 4 authors (26 links). The difference in year of publication may have contributed to the difference in the number of citations for Cyprus (for 2021) in comparison with Cameroon (for 2013).

#### 3.1.3. Analysis of Journals

**Figure 4:**
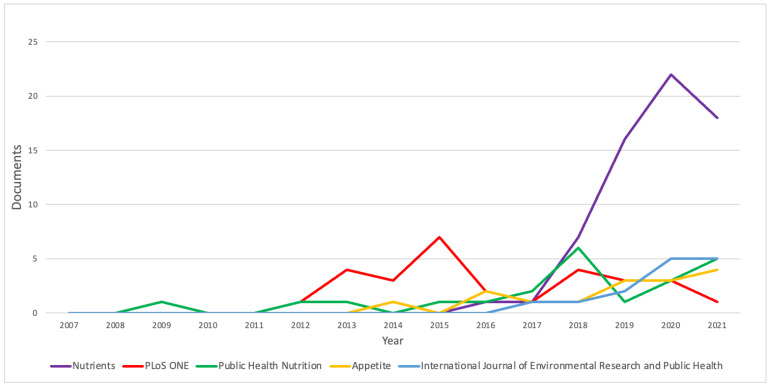
Analysis of journals.

Articles on AGEs in obesity have been published in 499 journals indexed in Scopus. *Nutrients* is the journal that accepts the greatest number of articles in this field, namely 65 articles (Figure 4). Other journals that are recorded as accepting large numbers of articles on AGEs up to January 2022 are *PLoS ONE*, *Public Health Nutrition*, *Appetite*, and *International Journal of Environmental Research and Public Health*, with 29, 22, 18, and 16 articles, respectively.

**Figure 5:**
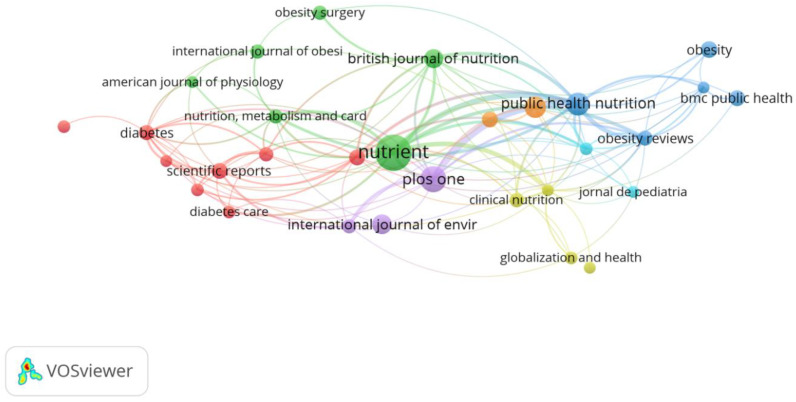
Bibliometric analysis of journals.

The VOSviewer analysis program was used to evaluate journals on the basis of the citations of their articles (Figure 5). There were 34 out of 599 sources of articles that met the minimum criterion of 5 documents and 30 sources of articles that had links with other sources of articles.

Open access journals may be defined as those containing documents that may be freely downloaded and read online without having subscriptions or filling their affiliations [27]. Our study shows that the majority or 60.9% (713/1170) of journals are open access. Table 3 shows that the median of the year of publication in open access journals is lower than in non-open access journals (*p* < 0.001); in other words, the articles are more recent. Our study shows that open access journals have higher medians for the number of citations than do non-open access journals (*p* < 0.05). The CiteScore has a negative association with the year of publication (r = −0.64 [95% CI: −0.67, −0.60], *p* <0.001).

Table 4, below, shows the top 15 journals based on the number of documents. The journal *Nutrients* contributed 5.6% of all documents on AGEs and obesity. The US is predominant in being the country with journal domains that publish the greatest number of articles on AGEs and obesity (seven journals), followed by the UK (five journals) and Europe (three journals).

#### 3.1.4. Most Frequently Cited Documents

Table 5 showed the 15 most-cited documents, representing 27.6% of the total number of citations.

#### 3.1.5. Collaboration between Authors

**Figure 6:**
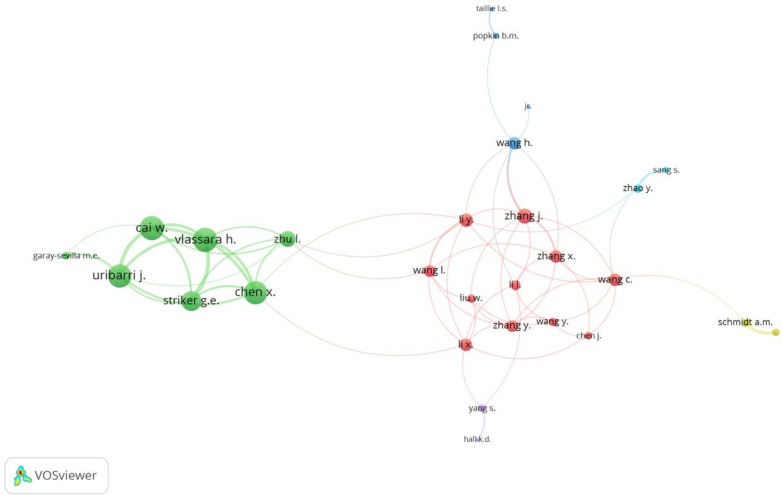
Author collaboration.

The collaboration between authors was evaluated using co-authorship by means of VOSviewer. There were 98 authors who were co-authors, among the 5954 authors having a minimum number of four documents. Figure 6 shows the 28 authors who collaborated with other authors in the writing of articles.

The collaboration of Cai, W., Vlassara, H., Uribarri, J., Striker, G.E., Chen, X., and Zhu, L. is most apparent as indicated by the proximity of the circles and the width of the lines connecting the circles. These authors have the same affiliation, namely Icahn School of Medicine at Mount Sinai, New York, NY, USA. Vlassara, H. and Cai, W. have the highest total link strengths of 19, followed by Uribarri, J. (18), Chen, X. (18), and Striker, G.E. (15). Chen, X. and Zhu, L. also collaborated with researchers outside their institution, as shown by the lines connecting the green and red clusters. The other authors in the red cluster collaborated more frequently with researchers in other clusters. This may be seen from the large number of lines connecting the red circles with circles of other colors, such as green, dark blue, light blue, and violet. Li, Y. is the topmost co-author (10 documents), followed by Uribarri, J. (9 documents), Schmidt, A.M. (8 documents), Popkin, B.M. (8 documents), and Vlassara, H. (7 documents). From the number of citations, there are five co-authors with the largest number of citations, namely Popkin, B.M. (2577), Vlassara, H. (844), Schmidt, A.M. (716), Uribarri, J. (628), and Cai, W. (520).

### 3.2. Analysis of Intellectual Structure

#### 3.2.1. Analysis of Keywords

Bibliometric analysis was used to evaluate the correlation of the most frequent keywords used by the authors of scientific articles on associated topics. This may facilitate the search for concepts and topics in the concerned field. This analysis used the VOSviewer application that can build a multidimensional representation of various topics. There are 53 keywords that appear minimally 10 times out of a total of 2505 keywords on this topic. The keywords are grouped into four clusters with a total of 440 links and a total link strength of 1434.

Obesity is the most popular keyword used by authors on this topic, and is found in Cluster 3 with 310 co-occurrences and has links with 50 other words. The next most frequently used keywords in Cluster 3 are physical activity, childhood obesity, food addiction, and community health problems. The keyword AGEs is the second most-used by several researchers in connection with this topic and lies in the red-colored Cluster 1. The green-colored Cluster 2 contains 18 nodes that are dominated by ultra-processed foods, whereas the yellow-colored Cluster 4 is focused on cardiovascular disease. (Table 6).

**Figure 7:**
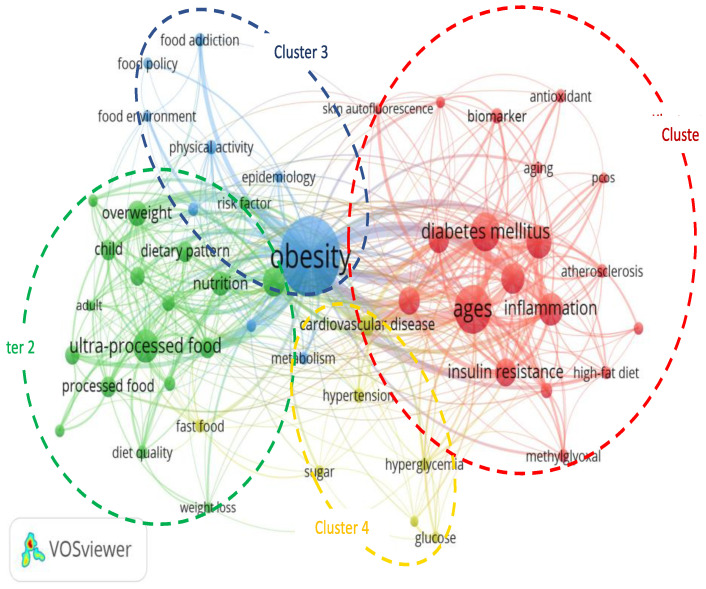
Visualization of the network of keywords used by authors, by co-occurrences.

Figure 7 shows that obesity is at the center of the keywords used by researchers and is closely associated with AGEs. The strength of the association between the keywords AGEs and obesity is shown by the thickness of the lines connecting the two circles (34 links). The use of AGEs as a keyword occurs together with the keywords oxidative stress (20 links), inflammation (15 links), insulin resistance (10 links), and type 2 diabetes mellitus (18 links). The topic of obesity is also frequently associated with ultra-processed foods (29 links). The circles of obesity and AGEs are seen to be closer to each other than to ultra-processed foods. The proximity of AGEs and AGE receptors is shown by the shorter distance between their circles (14 links). The topic of obesity is close to diet (34 links), which is associated with nutrition transition (12 links), quality (12 links), and dietary pattern, such as processed foods (19 links), ultra-processed foods (31 links), and fast foods (12 links).

#### 3.2.2. Co-Citation Networks of Cited Authors

There were 94,188 authors who became references in minimally two studies on AGEs and obesity, obtained through bibliographic analysis using VOSviewer, among whom 802 authors had 20 minimal citations. Table 7 below shows 15 of the most cited authors.

Monteiro, C.A. occupies the highest rank, with 746 citations, 111 links with other authors, and link strength of 54,339. This is followed by Popkin, B.M., with 517 citations, 182 links with other authors, and link strength of 34,295, while the third in line is Vlassara, H. with 495 citations, 192 links with other authors, and link strength of 58,763. Next come Schmidt, A.M., Cannon, G., Levy, R.B., Cai, W., Yamagishi, S., Moubarac, J.C., Uribarri, J., Thornalley, P.J., Takeuchi, M., Hu, F.B., Claro, R.M., and Brownlee, M., respectively.

**Figure 8:**
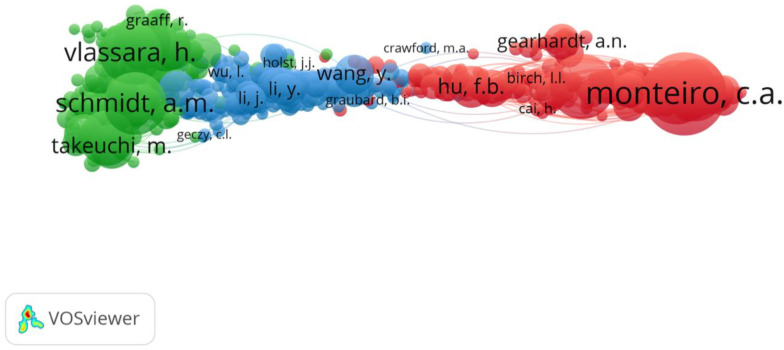
Co-citation networks of cited authors.

In Figure 8, the author co-citation map shows three clusters with a total of 144,439 links, and total link strength of 1,088,462. The first cluster consists of 289 authors, 14,623 citations, 89,267 links, and total link strength of 857,028. Monteiro, C.A. occupies the highest rank with 746 citations, 111 links with other authors, and link strength of 54,339, followed by Popkin, B.M., Cannon, G., and Levy, R.B. The second cluster consists of 268 authors, where the most prominent author is Schmidt, A.M. with 439 citations, 608 links, and total link strength of 29,721. Other authors with the highest number of co-citations in Cluster 2 are Vlassara, H., Cai, W., Yamagishi, S., and Uribarri, J. Cluster 3 consists of 55 authors, with Wang, Y. being the most prominent author in this cluster, having 193 citations, links to 192 other authors, with link strength of 8798. The next most prominent authors are Brownlee, M., Li, Y., and Zhang, Y.

#### 3.2.3. Co-Citation Source Networks

**Figure 9:**
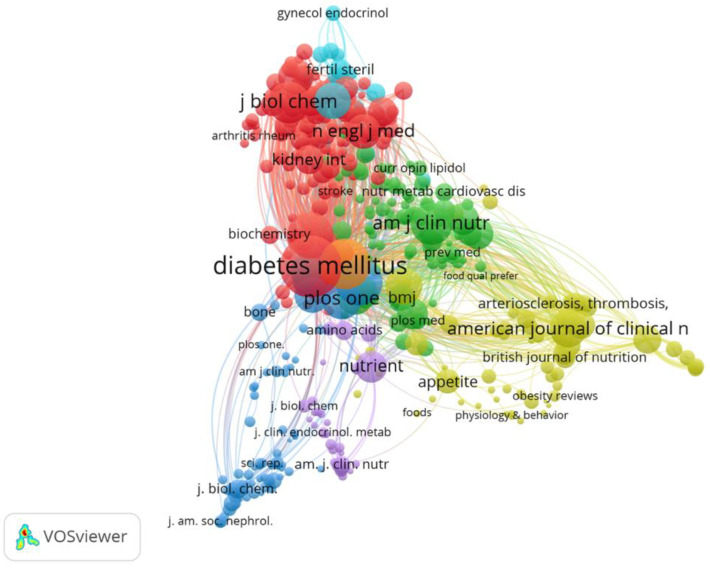
Co-citation source maps.

This analysis refers to direct observations on journals that have been repeatedly cited in AGE studies. The most active and influential scientific sources were identified using VOSviewer. There were 15,214 scientific sources in this study, and with the determined minimum threshold of 20 citations, we obtained 425 scientific sources that met the criteria for further discussion. The map below shows the results of the VOSviewer analysis, indicating the presence of six clusters in the network (Figure 9).

Table 8 below shows the top 15 scientific sources in AGE studies, which are the most-cited sources and have strong links with other scientific sources.

*Diabetes* occupies the top rank, with 1236 citations, 409 links with other scientific sources, and link strength of 30,087. The second rank is occupied by *Diabetes Care* with 845 citations, 420 links, and link strength of 20,139. *PLoS ONE* occupies the third rank, with 816 citations, 816 links, and link strength of 8106. The next ranking scientific sources are *The American Journal of Clinical Nutrition*, *Circulation*, *Nutrients*, *Diabetologia*, *The Lancet*, *Journal of Biological Chemistry*, *Nature*, *The Journal of Clinical Endocrinology and Metabolism*, *The New England Journal of Medicine*, *Atherosclerosis*, *The Journal of Clinical Investigation*, and *American Journal of Clinical Nutrition*, respectively.

Cluster 1 consists of 127 scientific sources, among which *Diabetes* is the most prominent, with 1236 citations, 409 links with other scientific sources, and link strength of 30,087. *The New England Journal of Medicine* and the *Journal of Biological Chemistry* are next in line. Cluster 2 consists of 102 sources, where *Nutrients* occupies first rank with the largest number of citations of 625, 209 links, and link strength of 3459.

Cluster 3 consists of 98 sources, with *Public Health Nutrition* being the most outstanding scientific source with 694 co-citations, 316 links, and link strength of 18,647. Cluster 4 consists of 64 sources, where *Appetite* is the scientific source having the highest number of co-citations in this cluster. Cluster 5 consists of 26 scientific sources, among which *The Journal of Clinical Endocrinology and Metabolism* is the first-ranking journal with the largest number of co-citations, whereas Cluster 6 has a single source, namely *Circulation* with 668 citations, 415 links, and link strength of 48,984.

## 4. Discussion

Our research showed there is an increase in references to AGEs in processed food publications especially from 2003 onwards. This increase in the number of publications may have been affected by several factors. The first is the increased prevalence of obesity in nearly all countries, both developed and developing countries [43,44]. Obesity is no longer limited to a certain socio-economic group [45]. Globalization, with changes in dietary patterns and sedentary lifestyles, both in urban and rural areas, also contributes to the occurrence of obesity. The second factor is the discovery of AGE pathways [29,46,47,48,49,50] and of databases of AGEs in foods from several countries [10,11,13,51,52]. The third factor is the open information system, where an increasing number of journals offer open access, [53,54] and the availability of big data to facilitate access to the most recent knowledge [55,56].

*Nutrients* is the journal that accepts the greatest number of articles in this field. The number of articles in *Nutrients* has increased sharply since 2018 with 7 documents, and continued to increase in 2019 and 2020 with, respectively, 16 and 22 documents per year. Although in 2021 the number of documents was 18, a decrease of 18.2% from the previous year, nevertheless this journal is still the largest contributor to the topics of AGEs and obesity. The decrease in the number of articles may have been caused by the current COVID-19 pandemic. The results of this study are in line with the study of Riccaboni, M. and Verginer, R., who showed a decrease in the number of non-COVID-19 publications by 10–12% during the pandemic in 2019 and 2020 [27].

In Figure 5, a thick line may be seen that connects *PLoS ONE* with *Public Health Nutrition* (link strength 12), showing the sufficiently high number of researchers preferring to publish articles in both the aforementioned journals. The node for *Nutrients* is adjacent to that of *PLoS ONE*, although differing in color. This may have been due to the fact that the accepted articles covered the same topics, although in general there is a difference in coverage between the two journals. The acceptance criteria in *Nutrients* are nutrition that is associated with human health and the methods for evaluating nutritional status, whereas *PLoS ONE* covers physics, medicine, engineering, social sciences, and the humanities. *Nutrients* has links to *Public Health Nutrition* (link strength 9) and *PLoS ONE* (link strength 5). This may be the referent in the selection of publications in similar research fields.

Our research showed the majority of journals are open access. A significant association of open access journal status with year of publication and number of citations exists. Several studies also show an increase in the number of open access journals [57,58]. Yang Li et al. also demonstrated a strong association between open access journals in medicine, biology, and science, based on the CiteScore [58]. It is known that the majority of articles on AGEs are in the subject area of medicine (Table 4). Citing articles takes time, as may be seen from the negative association between the CiteScore and year of publication (r = −0.64 [95% CI: −0.67, −0.60], *p* <0.001). The results of the present study agree with the study of Studenic, P. [59].

The *Diabetes* journal and the *British Journal of Nutrition* are the journals with the highest and second highest number of citations, namely 1080 and 1060 citations, respectively, although both journals are non-open access. This may have been caused by their age and ranking. *Diabetes* is known to have been in existence since 1952, with a q1 journal ranking and an h-index of 330. Similarly, the *British Journal of Nutrition* was established in 1947 and has a q1 journal ranking and an h-index of 188. *PLoS ONE* occupies the third highest rank for document citations with a total of 971 citations. *PLoS ONE* was founded in 2006; therefore, this journal may be said to be younger than *Diabetes* and the *British Journal of Nutrition*. In spite of this, the h-index of *PLoS ONE* (332) is the second highest after the *American Journal of Clinical Nutrition* (360). Apparently, in addition to journal age and impact factor, the open access status, quality, and the document topics [60] may play a role in determining the number of document citations.

The most frequently cited article is ‘Global nutrition transition and the pandemic of obesity in developing countries’, issued in 2012 by Popkin from the University of North Carolina, USA [28]. His article exposes the changes in lifestyle, i.e., an increased consumption of processed foods and decreased physical activity, which have attracted attention in low- and middle-income countries, both in urban and rural areas.

The hypothesis on the AGE pathways and the complications of type 2 diabetes was published by Evans, J. from the Medical Research Institute (J.L.E.), San Bruno, California, USA, in an article titled “Oxidative stress and stress-activated signaling pathways: A unifying hypothesis of type 2 diabetes” [29]. This document is the second most-cited one (1680 citations). The article ‘Dietary emulsifiers impact the mouse gut microbiota promoting colitis and metabolic syndrome’ is the third most-cited document [30]. Chassaing, B. discusses the metabolic syndrome induced by the emulsifiers carboxymethylcellulose and polysorbate-80, which were subsequently associated with changes in the species composition of the microbiota and the increase in pro-inflammatory potential [30].

Upon further analysis of these top 15 cited documents, it turns out that the most frequent topic is AGEs with noncommunicable disease (NCDs) complications, such as polycystic ovary syndrome (PCOS), diabetes mellitus, metabolic syndrome, or diabetic nephropathy [29,34,35,36,38,39,40]. The next most-cited topics are the increased consumption of processed foods and the effect of ultra-processed foods on obesity [28,31,32,37,42], and the association of AGEs with diet or of endogenous AGEs with inflammation [30,33,41].

Obesity is an abnormal condition due to an excess of fat in the body, which can be the cause of various pathological conditions. [1]. The fundamentally strong association between obesity and inflammatory mediators is caused by the chronic exposure to a high AGE diet, resulting in chronic inflammation and insulin resistance [61,62]. It was shown that binding between AGEs and receptor AGEs (membrane-bound receptors of AGE (mRAGE)) could cause cellular dysfunction interfering with cellular communication, changes in protein structure and function, and mitochondrial dysfunction, leading to cell death [63]. Binding to RAGE can also increase reactive oxygen species (ROS), activate inflammatory signaling such as the nuclear factor-k-light-chain-enhancer of activated β cells (NF-kβ), tumor necrosis factor-α (TNF-α), interleukin-6 (IL-6), and induce insulin resistance, which is often associated with obesity. Meanwhile, binding with other receptors such as AGE-R1, AGE-R2, and AGE-R3 plays a role in AGE clearance [64]. The close relationship between AGEs and the AGE receptors is also shown in several articles [29,39].

It has been observed that serum AGEs (ϵN-CML, methylglyoxal (MG)) are higher in patients with obesity and one other criterion of the metabolic syndrome than in patients without the metabolic syndrome criteria. This shows that obesity is correlated with a high AGE diet and not with a high calorie intake. Higher TNFα levels have also been reported in the same subjects, consistent with evidence that a high AGE level increases inflammation and insulin resistance [65]. Therefore, circulating AGEs have been suggested as biomarkers for the identification of the transition from obesity to metabolic syndrome, for detection of obesity at risk of metabolic syndrome, and for monitoring the efficacy of dietary interventions [66].

The close positive relationship between AGEs in foods and the level of calories consumed was reported by Baye et al., such that it is difficult to differentiate the individual contributions of each of these risk factors. Based on the tables and the database analysis included in the study paper, fats, meats, cheese, and pulses (if processed, canned, or grilled at high temperatures) have the highest AGE contents. The groups of fats and meats that have a high lipid and protein content tend to have higher AGE contents [9,67].

The study conducted by Uribarri also reported the same, in that although AGEs as a whole are not associated with markers of fat mass, in the presence of obesity and one or more criteria of metabolic syndrome, serum AGE levels are actually higher. Therefore, high AGE levels in obese adults may function as a signal for the transition from obesity to metabolic syndrome [68]. Bearing in mind that AGEs are modifiable non-traditional risk factors, these findings may assist in the timely and targeted prevention of the risk of metabolic syndrome [68,69].

Advanced glycation end products and inflammatory factors were not increased in subjects with obesity who did not have the other criteria of metabolic syndrome and were designated as being metabolically healthy, consistent with the view that an increase in body fat does not always indicate a risk for metabolic syndrome. In this context, the level of AGEs in healthy subjects with obesity may indicate a transition category of metabolic syndrome, such as high blood pressure, dyslipidemia, or subclinical cardiovascular disease, and determine the initial risk toward subsequent metabolic syndrome. Furthermore, outside of excessive nutrition, the exposure to AGE-rich foods may disclose important pointers and a temporal relationship between healthy and unhealthy obesity or obesity at risk for metabolic syndrome [68].

McGlashan, S.R. showed that a high fat diet has a positive correlation with AGEs in serum, tissues, and an increase in the total percentage of fat. High concentrations of AGEs in serum and tissues can be decreased with optimal physical activity intervention. In addition, reducing the intake of AGEs can also reduce the circulating levels of AGEs. From this study, it was found that a high-fat diet and physical activity affected the total body fat percentage, systemic inflammation, accumulation of AGEs, and increased the expression of proinflammatory cytokines. The effect of physical activity can increase the intra-hepatic breakdown process of AGEs that bind to proteins and peptides so that it will increase the elimination and reduction of AGEs in the kidneys [70,71].

Our study tried to identify the highly cited articles, journals, authors, countries, and the latest themes related to AGEs and obesity. Our study highlighted that there is an incremental increase in studies concerning ultra-processed food as part of a poor quality diet and relating to NCDs and obesity. There do not seem to be many data relating to ultra-processed food and the elderly. Therefore, there seems a need for research in this field. In the study of AGEs, procedure standardization in the laboratory between researchers is still needed.

One limitation of the present study is that the downloaded data may have been incomplete, because the extracted data were limited to titles and keywords, the Scopus database, published journal articles, and articles in English. There are other AGE structures that were not specifically written in the search terms such as N^ϵ^-(carboxyethyl)lysine (CEL), pentosidine, glyoxal (GO), and alpha-dicarbonyl compounds. Another limitation of this study is the possibility that other topics that are not related to this study were entered in the analysis and were visualized.

## 5. Conclusions

We present this bibliometric analysis to provide the most recent data on the description, visualization, and analysis of AGEs and obesity. There has been an increase in the study of processed foods that includes studies on AGEs and obesity. The United States are the largest contributors of studies in this field, with the highest total of citations. The *Nutrients* journal has published the largest number of articles on this topic, particularly in the last two years. The focus of the studies is currently centered on ultra-processed foods.

## Figures and Tables

**Table 1:** Distribution of top 15 countries by number of citations.

No.	Country	Region	Documents	Citations	Links	Total Link Strength
1	United States	America	382	19,362	103	55,724
2	Brazil	America	126	3611	94	27,028
3	United Kingdom	Europe	102	4203	98	27,961
4	Australia	Oceania	81	2551	100	18,628
5	Italy	Europe	78	3988	93	20,707
6	Germany	Europe	61	2715	94	16,838
7	Spain	Europe	58	2677	97	18,193
8	Japan	Asia	57	2028	68	7452
9	China	Asia	53	1145	87	6630
10	France	Europe	45	2337	94	12,203
11	Canada	America	44	1687	94	9564
12	Netherlands	Europe	38	2009	88	13,575
13	India	Asia	37	408	83	3124
14	South Korea	Asia	30	283	83	4140
15	Poland	Europe	29	342	69	2494

**Table 2:** Topmost ranking countries per cluster by number of documents.

Cluster	Documents	Citations	Links	Total Link Strength	Country
1	382	19,362	101	55,676	United States
Red	16	484	78	4551	New Zealand
(32 nodes)	16	457	82	4448	South Africa
2	57	2028	67	7451	Japan
Green	53	1145	87	6630	China
(15 nodes)	37	408	83	3124	India
3	78	3988	91	20,700	Italy
Blue	61	2715	92	16,835	Germany
(14 nodes)	14	168	60	1439	Egypt
4	24	648	24	648	Sweden
Yellow	11	101	11	101	Malaysia
(8 nodes)	6	48	6	48	Nigeria
5	58	2677	94	18,123	Spain
Violet	27	327	88	5631	Mexico
(7 nodes)	16	1626	74	3954	Israel
6	38	2009	87	13,574	Netherlands
Light Blue	22	821	81	11,929	Denmark
(7 nodes)	20	1408	73	3737	Greece
7	18	1369	78	5208	Belgium
Orange	15	517	65	1908	Portugal
(6 nodes)	3	1	47	883	Cyprus
8	81	2551	98	18,588	Australia
Brown	45	2337	92	12,116	France
(5 nodes)	1	302	26	467	Cameroon
9	126	3611	91	26,909	Brazil
Light Red	44	1687	94	9564	Canada
(4 nodes)	5	114	58	799	Hong Kong
10	102	4203	96	27,949	United Kingdom
Peach	1	1	4	237	Oman
(3 nodes)	1	1	4	237	South Sudan
11	12	180	86	5615	Ireland
Light Violet	7	254	58	3536	Romania
(3 nodes)	1	43	16	60	El Salvador

**Table 3:** Association of open access journal status with year of publication and number of citations (*n* = 1170).

Variable	Non-Open Access (*n* = 457)	Open Access (*n* = 713)	*p* Value
Year (in years)	2016 (2012–2020) ^‡,^**	2018 (2015–2020) ^‡,^**	<0.001
Citations	7 (2–23) ^‡,^*	11 (2–31) ^‡,^*	0.007

^‡^ Data are presented as median (25th percentile–75th percentile); Mann–Whitney test, statistically significant at * *p* < 0.05; and ** *p* < 0.001.

**Table 4:** Top 15 journals by number of documents.

Ranking	Journal	Print ISSN	E-ISSN	Country	Documents	% of Document Total	Citation	Q	h-Index	SJR (2020)	SNIP (2020)	Open Access (Feb 2022)
1	*Nutrients*	20726643		Switzerland	65	5.6	683	1	115	1.418	1.603	Yes
2	*PLoS ONE*	19326203		United States	29	2.5	971	1	332	0.990	1.349	Yes
3	*Public Health Nutrition*	13689800	14752727	United Kingdom	22	1.9	843	1	137	1.166	1.368	Yes
4	*Appetite*	1956663	10958304	United States	18	1.5	174	1	144	1.131	1.448	Yes
5	*International Journal of Environmental Research and Public Health*	16617827	16604601	Switzerland	16	1.4	39	2	113	0.747	1.356	Yes
6	*British Journal of Nutrition*	71145	14752662	United Kingdom	15	1.3	1060	1	188	1.073	1.194	No
7	*Obesity*	19307381		United States	11	0.9	372	1	199	1.438	1.356	No
8	*BMC Public Health*	14712458		United Kingdom	10	0.9	118	1	143	1.230	1.656	Yes
9	*Medical Hypotheses*	3069877	15322777	United States	10	0.9	324	3	87	0.441	0.530	Yes
10	*European Journal of Nutrition*	14366207	14366215	Germany	9	0.8	218	1	96	1.321	1.532	No
11	*Scientific Reports*	20452322		United Kingdom	9	0.8	70	1	213	1.240	1.377	Yes
12	*Diabetes*	121797		United States	8	0.7	1080	1	330	3.219	1.901	No
13	*Obesity Reviews*	14677881	1467789X	United Kingdom	8	0.7	613	1	162	2.845	3.056	Yes
14	*American Journal of Clinical Nutrition*	29165	19383207	United States	7	0.6	360	1	336	2.608	2.294	Yes
15	*Clinical Nutrition*	2615614	15321983	United States	7	0.6	49	1	140	1.915	2.517	No

Abbreviations: ISSN: International Standard of Serial Number; E-ISSN: Electronic International Standard of Serial Number; SJR: Scimago journal & country rank; SNIP: Source Normalized Impact per Paper.

**Table 5:** Top ranks for citations of documents on AGEs and obesity.

No.	Authors	Year	Title	Number of Citations	Reference Number
1	Popkin, B.M.	2012	*Global nutrition transition and the pandemic of obesity in developing countries*	2189	[28]
2	Evans, J.L.	2002	*Oxidative Stress and Stress-Activated Signaling Pathways: A Unifying Hypothesis of Type 2 Diabetes*	1680	[29]
3	Chassaing, B.	2015	*Dietary emulsifiers impact the mouse gut microbiota promoting colitis and metabolic syndrome*	902	[30]
4	Bibbins-Domingo, K.	2010	*Projected effect of dietary salt reductions on future cardiovascular disease*	889	[31]
5	World Health Organization	2003	*Diet, nutrition and the prevention of chronic diseases*	796	[32]
6	Calder, P.C.	2011	*Dietary factors and low-grade inflammation in relation to overweight and obesity*	600	[33]
7	Wada, J.	2013	*Inflammation and the pathogenesis of diabetic nephropathy*	430	[34]
8	Fiorentino, T.V.	2013	*Hyperglycemia-induced Oxidative Stress and its Role in Diabetes Mellitus Related Cardiovascular Diseases*	401	[35]
9	Tanji, N.	2000	*Expression of advanced glycation end products and their cellular receptor RAGE in diabetic nephropathy and nondiabetic renal disease*	390	[36]
10	Hall, K.D.	2019	*Ultra-Processed Diets Cause Excess Calorie Intake and Weight Gain: An Inpatient Randomized Controlled Trial of Ad Libitum Food Intake*	368	[37]
11	Conway, G.	2014	*The polycystic ovary syndrome: a position statement from the European Society of Endocrinology*	364	[38]
12	Donath, M.Y.	1999	*Hyperglycemia-induced beta-cell apoptosis in pancreatic islets of Psammomys obesus during development of diabetes.*	352	[39]
13	Scuteri, A.	2004	*Metabolic syndrome amplifies the age-associated increases in vascular thickness and stiffness*	348	[40]
14	Lontchi-Yimagou, E.	2013	*Diabetes Mellitus and Inflammation*	302	[41]
15	Louzada, M.L.C.	2015	*Consumption of ultra-processed foods and obesity in Brazilian adolescents and adults*	294	[42]

**Table 6:** Most frequently used keywords by authors.

Cluster	Co-Occurrences	Links	Total Link Strength	Author Keyword
1 Red (19 nodes)	130	33	195	AGEs
	86	24	132	Diabetes Mellitus
	66	26	119	Oxidative stress
	65	23	122	Inflammation
	58	24	88	Age receptor
	51	25	101	Insulin resistance
	51	34	103	Metabolic syndrome
	46	23	62	Diabetes Mellitus, type 2
	18	10	31	Biomarker
	18	18	37	Glycation
	14	8	17	Antioxidant
	14	13	25	Atherosclerosis
	14	11	25	Methylglyoxal
	13	7	11	Diabetic nephropathy
	12	13	28	High-fat diet
	11	10	16	Aging
	11	14	25	Bariatric surgery
	10	10	22	PCOS
	10	8	18	Skin autofluorescence
2 Green (18 nodes)	68	31	105	Ultra-processed food
	55	34	107	Diet
	42	24	83	Overweight
	40	27	74	Nutrition
	30	20	47	Child
	29	22	57	Adolescent
	29	18	37	Dietary pattern
	29	19	43	Processed food
	26	12	33	Nutrition transition
	25	20	44	Body mass index
	19	16	41	Food consumption
	16	11	28	Food processing
	15	12	28	Diet quality
	14	14	32	Risk factor
	13	10	24	Food
	12	6	16	Non-communicable diseases
	10	13	20	Adult
	10	8	14	Weight loss
3 Blue (9 nodes)	310	50	517	Obesity
	16	17	26	Physical activity
	15	10	13	Childhood obesity
	15	6	18	Food addiction
	13	15	25	Public health
	12	10	20	Metabolism
	11	15	22	Epidemiology
	11	10	16	Food environment
	11	3	5	Food policy
4 Yellow (7 nodes)	30	27	75	Cardiovascular disease
	16	13	22	Hypertension
	15	12	22	Fast food
	13	15	28	Hyperglycemia
	11	7	17	Glucose
	11	10	16	Sugar
	10	9	16	Fructose

**Table 7:** Top 15 Co-citations of authors in references of AGEs and obesity.

Ranking	Authors	Co-citations	Links	Total Link Strength
1	Monteiro, C.A.	737	213	54,339
2	Popkin, B.M.	517	276	34,295
3	Vlassara, H.	495	269	58,763
4	Schmidt, A.M.	494	237	47,118
5	Cannon, G.	464	205	33,401
6	Levy, R.B.	445	204	32,228
7	Cai, W.	392	270	44.311
8	Yamagishi, S.	369	239	32,718
9	Moubarac, J.C.	333	195	25,603
10	Uribarri, J.	319	245	31,732
11	Thornalley, P.J.	287	226	58,303
12	Takeuchi, M.	247	237	24.424
13	Hu, F.B.	242	296	23,864
14	Claro, R.M.	230	181	15,166
15	Brownlee, M.	212	277	27,471

**Table 8:** Top 15 co-citations of scientific sources.

Ranking	Source	Citations	Links	Total Link Strength
1	*Diabetes*	1236	409	30,087
2	*Diabetes Care*	845	420	20,139
3	*PLoS ONE*	816	816	8106
4	*The American Journal of Clinical Nutrition*	674	123	9349
5	*Circulation*	668	415	14,980
6	*Nutrients*	625	209	3459
7	*Diabetologia*	580	403	17,875
8	*The Lancet*	571	73	8304
9	*Journal of Biological Chemistry*	435	194	13,240
10	*Nature*	431	169	8228
11	*The Journal of Clinical Endocrinology and Metabolism*	421	174	7334
12	*The New England Journal of Medicine*	364	335	9194
13	*Atherosclerosis*	328	398	9055
14	*The Journal of Clinical Investigation*	322	230	9765
15	*American Journal of Clinical Nutrition*	182	199	7733

## Data Availability

The analyzed dataset is in the Supplementary Materials.

## Supplementary Materials

The following supporting information can be downloaded at: https://www.mdpi.com/article/10.3390/nu14245255/s1, Table S1: The analyzed dataset.
